# miR-29a Negatively Affects Glucose-Stimulated Insulin Secretion and MIN6 Cell Proliferation via Cdc42/*β*-Catenin Signaling

**DOI:** 10.1155/2019/5219782

**Published:** 2019-08-28

**Authors:** Jing Duan, Xian-Ling Qian, Jun Li, Xing-Hua Xiao, Xiang-Tong Lu, Lin-Chen Lv, Qing-Yun Huang, Wen Ding, Hong-Yan Zhang, Li-Xia Xiong

**Affiliations:** ^1^Department of Pathophysiology, Medical College, Nanchang University, 461 Bayi Road, Nanchang 330006, China; ^2^Department of Pathology, Second Affiliated Hospital, Nanchang University, No. 1 Mingde Road, Nanchang 330006, China; ^3^Department of Burn, The First Affiliated Hospital, Nanchang University, 17 Yongwaizheng Road, Nanschang 330066, China

## Abstract

**Background:**

Diabetes is a progressive metabolic disease characterized by hyperglycemia. Functional impairment of islet *β* cells can occur to varying degrees. This impairment can initially be compensated for by proliferation and metabolic changes of *β* cells. Cell division control protein 42 (Cdc42) and the microRNA (miRNA) miR-29 have important roles in *β*-cell proliferation and glucose-stimulated insulin secretion (GSIS), which we further explored using the mouse insulinoma cell line MIN6.

**Methods:**

Upregulation and downregulation of miR-29a and Cdc42 were accomplished using transient transfection. miR-29a and Cdc42 expression was detected by real-time PCR and western blotting. MIN6 proliferation was detected using a cell counting kit assay. GSIS under high-glucose (20.0 mM) or basal-glucose (5.0 mM) stimulation was detected by enzyme-linked immunosorbent assay. The miR-29a binding site in the Cdc42 mRNA 3′-untranslated region (UTR) was determined using bioinformatics and luciferase reporter assays.

**Results:**

miR-29a overexpression inhibited proliferation (*P* < 0.01) and GSIS under high-glucose stimulation (*P* < 0.01). Cdc42 overexpression promoted proliferation (*P* < 0.05) and GSIS under high-glucose stimulation (*P* < 0.05). miR-29a overexpression decreased Cdc42 expression (*P* < 0.01), whereas miR-29a downregulation increased Cdc42 expression (*P* < 0.01). The results showed that the Cdc42 mRNA 3′-UTR is a direct target of miR-29a *in vitro*. Additionally, Cdc42 reversed miR-29a-mediated inhibition of proliferation and GSIS (*P* < 0.01). Furthermore, miR-29a inhibited *β*-catenin expression (*P* < 0.01), whereas Cdc42 promoted *β*-catenin expression (*P* < 0.01).

**Conclusion:**

By negatively regulating Cdc42 and the downstream molecule *β*-catenin, miR-29a inhibits MIN6 proliferation and insulin secretion.

## 1. Introduction

Diabetes is a progressive metabolic disease characterized by hyperglycemia, and it is the third most common chronic disease worldwide, after cancer and cardiovascular disease [1, 2]. Based on the pathogenesis of diabetes, it can be divided into type 1 diabetes mellitus (T1DM) and type 2 diabetes mellitus (T2DM) [3]. T1DM is characterized by autoimmune-induced loss of *β* cells in the pancreas, which leads to insufficient insulin secretion or complete insulin deficiency [4]. T2DM is caused by genetic, environmental, behavioral, and other risk factors, and it is characterized by hyperglycemia, insulin resistance, and relative insulin deficiency [5]. During the development of both T1DM and T2DM, functional impairment of islet *β* cells can occur to varying degrees [6]. This impairment can initially be compensated for by *β*-cell proliferation and changes in metabolism. However, as the disease progresses, the islet *β*-cell proliferation is reduced and insulin secretion continues to decline, eventually leading to irreversible functional failure. Therefore, studying islet *β*-cell proliferation and insulin secretion is of great significance.

It has been reported that both islet *β* cells self-replication under elevated blood glucose conditions and transformation of islet *α* cells to *β* cells may increase the number of islet *β* cells [7–9]. In *β* cells, glucose can regulate insulin secretion in a process known as glucose-stimulated insulin secretion (GSIS) [10]. GSIS can maintain blood glucose levels within the physiological range, which involves transportation of glucose into *β* cells through the plasma membrane glucose transporters, followed by transformation of glucose to glucose-6-phosphate and the subsequent rises of Ca^2+^ and metabolic coupling factors such as ATP, glutamate, NADPH, and monoacylglycerol from glycolytic or mitochondrial metabolism [11, 12]. GSIS is composed of two phases: a rapid and transient first phase and a slow and lasting second phase. Both phases involve active mobilization of insulin secretory granules from the cytoplasm to the plasma membrane, requiring small GTP-binding proteins known as small GTPases-mediated actin cytoskeletal remodeling [12–14].

MicroRNAs (miRNAs) are short non-coding RNAs of approximately 22-nt in length, which are recognized as important regulators of gene expression after transcription [15]. To date, the human genome has been shown to encode more than 2000 miRNAs, which are involved in a wide variety of biological and pathological processes [16]. miRNAs act as negative regulators by repressing mRNA translation or causing mRNA degradation after transcription, so abnormal miRNA expression interferes with many physiological and pathological processes [17]. Many miRNAs have been found to be involved in the pathogenesis of diabetes and insulin resistance, and they affect the function of islet *β* cells [18, 19]. miR-29a is one of the most abundant miRNAs expressed in the *β* cells of the mouse and human pancreas, and many studies have shown upregulation of miR-29a in diabetic models [20–22]. It belongs to the miR-29 family, which is composed of three closely related precursors: miR-29a, miR-29b1, and miR-29b2 (which are identical but encoded by two distinct precursor stem sequences), and miR-29c [23]. The sequences of mature miR-29 family members are conserved in humans, rats, and mice, and the seed sequence that regulates gene expression by binding to target mRNAs, AGCACC, is also identical [20]. miR-29a has been reported to play a negative regulatory role in insulin secretion by human and mouse islet *β* cells, and miR-29a overexpression reduced GSIS levels *in vitro* [24]. Conversely, it has also been reported that miR-29a positively regulates insulin secretion *in vivo* [20]. Therefore, the role of miR-29a in GSIS warrants further study.

Cell division control protein 42 (Cdc42) is a member of the Rho family of small GTPases [25], and it plays an important role in the second phase of GSIS [26, 27]. It has been confirmed that Cdc42 can be found in cloned islet *β* cells, normal mouse islet cells, and normal human islet cells, and it is localized to insulin secretory granules [28]. Under physiological conditions, glucose regulates actin cytoskeleton rearrangement and stimulates insulin secretion by mediating the transformation between Cdc42-GDP (inactive) and Cdc42-GTP (active) [29]. Salunkhe et al. found that phosphorylation of focal adhesion kinase (FAK), which phosphorylates Cdc42 under glucose stimulation, disrupts the F-actin barrier, allowing insulin secretory granules to redistribute in islet *β* cells and thereby promoting insulin secretion [30]. It has also been reported that Cdc42 mediates insulin secretory granule transportation and insulin secretion via the PAK1-Raf-1/MEK/ERK pathway [31]. Additionally, Cdc42-PAK1-Rac1 has been shown to play a regulatory role in insulin exocytosis and may also play a role in actin remodeling and insulin granule mobilization [32]. These studies suggest that Cdc42 has a significant role in GSIS.

*β*-Catenin is a transcription factor, mostly known as a key component of the canonical Wnt signaling pathway to regulate cell proliferation [33, 34]. Activated Wnt signaling inhibits ubiquitin-mediated proteasomal degradation of *β*-catenin, thus causing *β*-catenin to accumulate. Subsequently, *β*-catenin translocates to the nucleus to form a transcriptionally active complex with T-cell factor (TCF) and lymphoid enhancer factor and promotes transcription of proliferation-related genes, such as c-Myc [35, 36]. *β*-Catenin can also regulate cell-cell adhesion between pancreatic *β* cells via forming complexes with cadherins, which is important for correct regulation of insulin release [37–39]. Emerging evidence has shown upregulation of *β*-catenin protein under diabetic conditions, and hyperglycemia can promote translocation of *β*-catenin [40, 41].

In a study on human non-small cell lung cancer, miR-29a overexpression led to significant inhibition of Cdc42 protein expression, whereas Cdc42 mRNA expression was unchanged [42]. In gastric cancer, miR-29a inhibits Cdc42 expression at both the protein and RNA levels [43]. Additionally, miR-29a inhibits glioma invasion by targeting Cdc42 [44]. Furthermore, in breast cancer, Cdc42 negatively regulates p53, and miR-29a positively regulates p53 by targeting Cdc42 and, notably, miR-29a inhibits insulin secretion by negatively regulating Cdc42 and P85 [45]. Cdc42 has also been identified as a direct target of miR-29a in mouse osteoclasts using a luciferase reporter assay [46]. And many studies have confirmed that *β*-catenin can be regulated as a downstream molecule of Cdc42 [47–49]. Therefore, the role of miR-29a in islet *β*-cell proliferation and GSIS may be achieved through interaction with Cdc42/*β*-catenin signaling.

The aim of the current study was to explore the effects of miR-29a and Cdc42 on islet *β*-cell proliferation and GSIS using MIN6 cells, and to identify the effect of the miR-29a/Cdc42/*β*-catenin signaling cascade in these cells. The results indicate that miR-29a plays a negative regulatory role in GSIS and MIN6 cell proliferation, whereas Cdc42 plays a positive regulatory role. And miR-29a negatively affects GSIS and MIN6 cell proliferation via inhibiting Cdc42/*β*-catenin signaling pathway.

## 2. Materials and Methods

### 2.1. Cell Line and Culture

The mouse insulinoma cell line MIN6 was obtained from BoGu Biotechnology Co. Ltd. (Shanghai, China). High-glucose (4500 mg/L) Dulbecco's modified Eagle's medium (DMEM) was purchased from Hyclone (Logan, UT, USA). Fetal bovine serum (FBS) was purchased from Biological Industries (Cromwell, CT, USA). The MIN6 cells were maintained in high-glucose DMEM supplemented with 12% FBS, 10 *μ*l/L *β*-mercaptoethanol (Sigma-Aldrich, St. Louis, MO, USA), 100 U/ml penicillin, and 100 *μ*g streptomycin mixture (Solarbio, Beijing, China) at 37°C in 5% CO_2_.

### 2.2. Transient Transfection

5.5 × 10^5^ MIN6 cells were inoculated in 6-well plate and incubated for 24 hours in DMEM medium. The 20 *μ*M final concentration of miR-29a mimic, inhibitor, negative control (NC; miR-29a NC), and Cdc42-pcDNA3.1 was synthesized by Gemma Co. Ltd. (Shanghai, China). siRNA fragments (siRNA-497, siRNA-569, and siRNA-643) and an NC-siRNA fragment were also obtained from Gemma Co. Ltd. Oligonucleotide and plasmid transfection was conducted using Lipofectamine 2000 (Gemma Co. Ltd.). Opti-MEM was purchased from Gibco company (Grand Island, NY, USA). Firstly, 100 pmol of siRNA was added to 200 *μ*l Opti-MEM and blended gently. Secondly, 200 *μ*l Opti-MEM was used to dilute 5 *μ*l lip2000 reagent. This was maintained for 5 minutes at room temperature after mixing. The lip2000 reagent diluent was then added to the siRNA diluent at room temperature for 20 minutes to form the siRNA-lip2000 complex. The medium was replaced by serum-free medium, and siRNA-lip2000 complex was added into the pore containing cells and medium. The fluorescence and cell status were observed after 6 hours. The serum-free medium was extracted and medium was added. The sequences of the oligonucleotides are shown in Table 1. After 24–48 h of transfection, MIN6 cells were used for the following experiments.

### 2.3. Real-Time Polymerase Chain Reaction (RT-PCR)

When the cell confluency reached 75%, the miR-29a mimic, miR-29a inhibitor, and miR-29a-NC, NC-siRNA, siRNA-497, siRNA-569 and siRNA-643 were separately transiently transfected into MIN6 cells. After 36 h, total RNA was extracted from the MIN6 cells using the total RNA isolation reagent (Omega, Norcross, GA, USA) according to the manufacturer's instructions. 2 *μ*l PrimeScript buffer, 0.5 *μ*l Random 6 mers, 0.5 *μ*l Oligo dT Primer, 0.5 *μ*l 1 × PrimeScript RT Enzyme Mix I, 0.5 *μ*l gene-specific primers, up to 10 *μ*l RNase free ddH_2_O, and 500 ng total RNA were added to prepare for reverse transcription system. Then, reverse transcription was performed at 37°C for 15 minutes, and after 5 seconds at 85°C, the machine was maintained at 4°C (when using gene-specific primers, the first step of reverse transcription reaction condition was changed to 42°C 15 minutes). RT-PCR was performed with an ABI StepOnePlus Real-Time PCR System (Applied Biosystems, Foster City, CA, USA). 0.4 *μ*l of forward primer, 0.4 *μ*l of reverse primer, 10 *μ*l of TB Green Premix Ex Taq, 0.4 *μ*l 50 × ROX reference dye, 2 *μ*l of template, 6.8 *μ*l of ddH_2_O were added to form 20 *μ*l of the total reaction system. After mixing, 18 *μ*l total reaction system and 2 *μ*l cDNA were added to each pore. The reaction mixture was incubated at 95°C for 30 s followed by 40 cycles of 5 s at 95°C and 30 s at 60°C. The 2^−ΔΔCT^ method was used to calculate the relative difference in gene expression.

### 2.4. Western Blot Analysis

Transient transfection was performed when the MIN6 cell confluency reached 75%, and protein extraction was carried out after 40 h. Cdc42-pcDNA3.1, Cdc42-siRNAs, miR-29a mimic, miR-29a inhibitor, and miR-29a-NC were transiently transfected into cells. Protein was electrophoresed on SDS-polyacrylamide gel consisting of 5% stacking gel and 12% separating gel (Solarbio). First, 15 *μ*g of protein was added to each slot. After the proteins were separated, they were transferred to a polyvinylidene fluoride (PVDF) membrane (Merck Millipore, Billerica, MA, USA) under 200 mA for 1 hour and 15 minutes. Next, 5% nonfat milk (Becton, Dickinson, and Company, Franklin Lakes, NJ, USA) was used for 2 h blocking. Then, the PVDF membrane was incubated overnight at 4°C with diluted (1 : 1000) primary antibodies (Cdc42 antibody, Abcam, Cambridge, MA, USA; *β*-catenin, Affinity Biologicals, Shanghai, China; and *β*-actin, Zhongshanjinqiao Company, Beijing, China). Subsequently, the membrane was incubated for 1.5 h at room temperature with diluted (1 : 5000) secondary antibodies (HPR-labeled anti-rabbit IgG of goat, HPR-labeled anti-rat IgG of goat; both were purchased from Zhongshanjinqiao Company). Lastly, the proteins were detected using an EasySee Western Blot Kit (TransGen Biotech, Beijing, China) with a Gel Imaging System (Bio-Rad, Hercules, CA, USA).

### 2.5. Cell Proliferation Assay

A cell proliferation assay was performed using a cell counting kit (CCK; TransGen) after Cdc42-pcDNA3.1, Cdc42-siRNA-643, miR-29a inhibitor + Cdc42-siRNA-643, and miR-29a mimic + Cdc42-pcDNA3.1 were separately transiently transfected into MIN6 cells. MIN6 cells were incubated in 96-well plates for 24 h. Next, 10 *μ*l CCK solution and 90 *μ*l high-glucose (4500 mg/L) DMEM were added to the cells. The cells were then placed in an incubator at 37°C for 1 h before assessment. The optical density at 450 nm (OD_450_) at 24, 48, and 72 h was measured using an SpectraMax Paradigm enzyme labelling apparatus (Molecular Devices LLC, Sunnyvale, CA, USA), and corresponding cell growth curves were plotted.

### 2.6. Insulin Secretion Assay

After 40 h of transfection, the medium was removed and cells in each group were divided into two subgroups. Next, 1 ml Krebs-Ringer bicarbonate HEPES (KRBH, PanEra, Guangzhou, China) buffer was added to each well and the mixture was incubated for 1 h. Thereafter, the KRBH buffer was removed and 1 ml KRBH containing 5.0 or 20.0 mM glucose (Solarbio) was added into subgroups separately for 1 h. The levels of insulin were detected by enzyme-linked immunosorbent assay (ELISA) using an ELISA Kit for Insulin (Cloud-Clone Corp., Wuhan, China) according to the manufacturer's instructions.

### 2.7. Luciferase Reporter Assays

The miR-29a binding site in the Cdc42 mRNA 3′-UTR was identified in a bioinformatics analysis (Gemma Co. Ltd.), and a luciferase reporter assay was performed. First, the entire nonmutated 3′-untranslated region (UTR) of the Cdc42 gene was cloned into a pGL3-Basic vector (Gemma Co. Ltd.) at a site immediately downstream of the luciferase gene. Second, the Cdc42 3′-UTR was mutated with a mutagenesis kit (Promega, Madison, WI, USA) and similarly cloned into a pGL3-Basic vector. 1 × 10^5^ MIN6 cells were seeded into 6-well plates and cultured for 24 h. Next, the cells were cotransfected with 2.5 *μ*g of either of the pGL3-Basic vectors, and 2.5 *μ*g of either miR-29a or miR-29a-NC using Lipofectamine 2000 (Gemma Co. Ltd.). At 48 h after transfection, cell lysates were prepared using Luciferase Assay Buffer II, and the luciferase activity was measured using a Luciferase Assay System (Promega). The experiment was performed in triplicate.

### 2.8. Statistical Analysis

Statistical analysis was performed using Prism 6 (GraphPad Software, San Diago, CA, USA) or SPSS 17.0 (SPSS Inc., Chicago, IL, USA). All data are presented as mean ± standard deviation (SD). One-way analysis of variance (ANOVA) and Student's *t*-test were used to assess the differences between groups. *P* < 0.05 and *P* < 0.01 were considered to be statistically significant and highly statistically significant, respectively.

## 3. Results

### 3.1. Effects of miR-29a on MIN6 Cells

#### 3.1.1. Transfection Efficiency of miR-29a Mimic and Inhibitor

**Figure 1:**
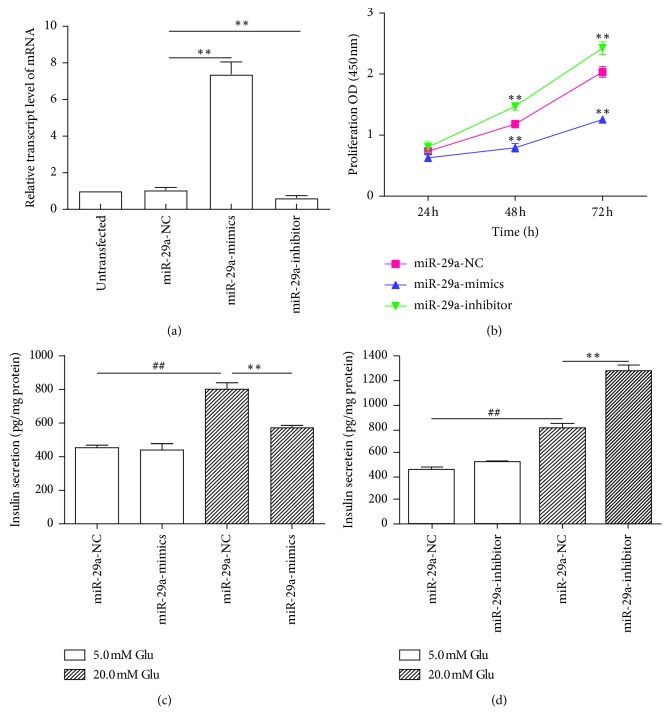
Effects of miR-29a on MIN6 cells. (a) Effects of miR-29a mimic and inhibitor on miR-29a mRNA. RT-PCR to detect miR-29a mRNA expression after transient transfection with miR-29a mimic and inhibitor (*n* = 3). ^*∗∗*^*P* < 0.01, compared with miR-29a NC group, as assessed by paired Student's *t*-test. (b) Effects of miR-29a on MIN6 cell proliferation. CCK assay to detect proliferation after transfection with miR-29a mimic and inhibitor (*n* = 3). ^*∗∗*^*P* < 0.01, compared with miR-29a NC group, as assessed by one-way ANOVA, followed by Fisher's least significant difference test. (c and d) Effects of miR-29a on insulin secretion by MIN6 cells. ELISA to detect insulin secretion levels in MIN6 cells after transient transfection with miR-29a mimic and inhibitor under basal-glucose (5.0 mM) and high-glucose (20.0 mM) stimulation (*n* = 3). ^##^*P* < 0.01, compared with 5.0 mM glucose group, and ^*∗∗*^*P* < 0.01, compared with miR-29a-NC group, as assessed by paired Student's *t*-test. Data are shown as mean ± SD. NC: negative control.

To ensure the validity of subsequent miR-29a-related experiments, we determined the transfection efficiency of the miR-29a mimic and inhibitor. The miR-29a mRNA transcription level significantly increased in the miR-29a mimic group compared with the miR-29a-NC group (*P* < 0.01) and significantly decreased in the miR-29a inhibitor group (*P* < 0.01) (Figure 1(a)). These results indicated successful transfection.

#### 3.1.2. miR-29a Negatively Effects MIN6 Cell Proliferation

To determine the effect of miR-29a on MIN6 cell proliferation, we increased and decreased miR-29a expression using the miR-29a mimic and inhibitor, respectively, and detected the proliferation rate at 24, 48, and 72 h. The CCK results showed that there were no significant differences in the proliferation rate between the miR-29a NC group and the miR-29a mimic and inhibitor groups after 24 h. In contrast, the proliferation rate of the miR-29a mimic group significantly decreased after 48 h (*P* < 0.01) and 72 h (*P* < 0.01), and the proliferation rate in the miR-29a inhibitor group significantly increased after 48 h (*P* < 0.01) and 72 h (*P* < 0.01) (Figure 1(b)). These results indicated that miR-29a negatively effects MIN6 cell proliferation.

#### 3.1.3. miR-29a Negatively Effects Insulin Secretion by MIN6 Cells

To identify the effect of miR-29a on insulin secretion by MIN6 cells, we increased and decreased miR-29a expression using the miR-29a mimic and inhibitor, respectively, and detected the level of insulin secretion after stimulation with 5.0 and 20.0 mM glucose. The ELISA results showed that miR-29a overexpression inhibited insulin secretion under high-glucose stimulation (*P* < 0.01) (Figure 1(c)), and miR-29a downregulation promoted insulin secretion under high-glucose stimulation (*P* < 0.01) (Figure 1(d)). Regardless of whether miR-29a was up- or downregulated, there was no effect on insulin secretion under basal-glucose stimulation (Figures 1(c) and 1(d)). These results indicated that miR-29a plays a negative regulatory role in GSIS, but not in insulin secretion at physiological blood glucose levels.

### 3.2. Effects of Cdc42 on MIN6 Cells

#### 3.2.1. Transfection Efficiency of Cdc42-pcDNA3.1

**Figure 2:**
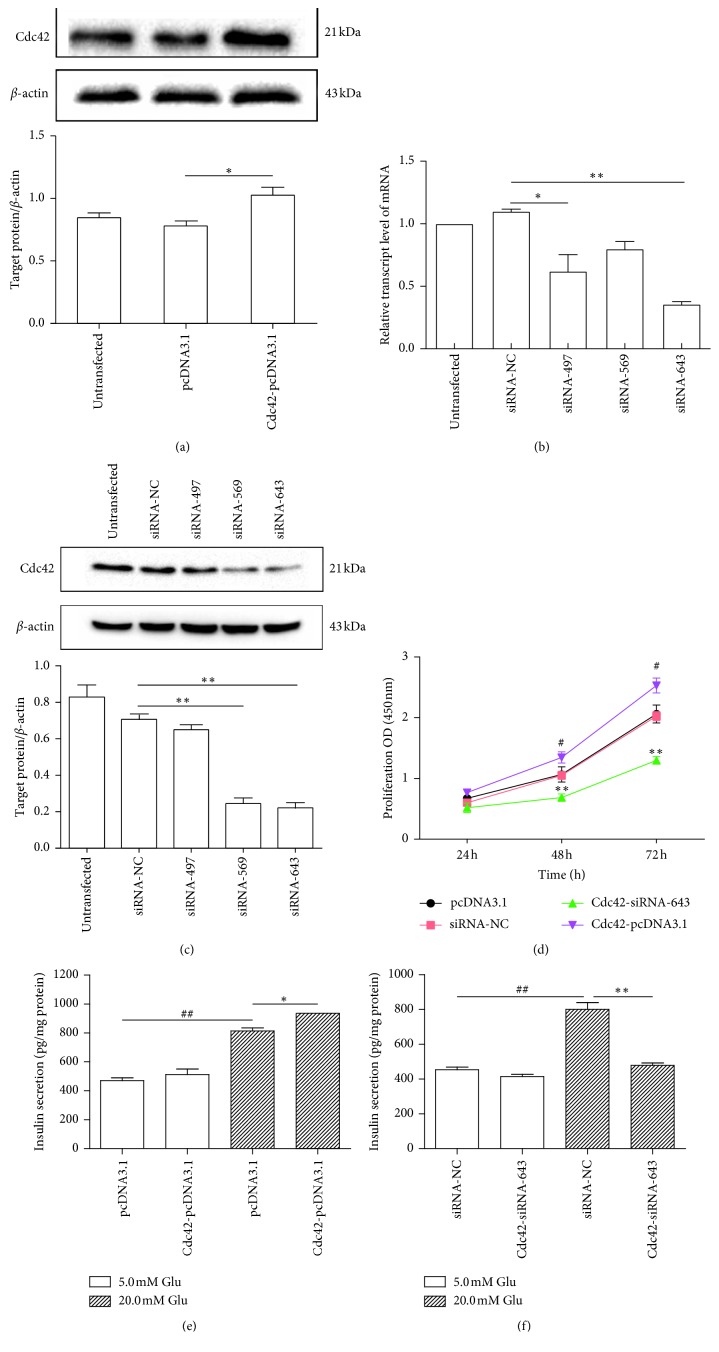
Effects of Cdc42 on MIN6 cells. (a) Effects of Cdc42-pcDNA3.1 on Cdc42 expression in MIN6 cells. Western blot to detect Cdc42 protein expression after transfection with Cdc42-pcDNA3.1 (*n* = 3). ^*∗*^*P* < 0.01, compared with pcDNA3.1 group, as assessed by paired Student's *t*-test. (b and c) Screening of Cdc42-siRNA fragments. (b) RT-PCR to detect Cdc42 mRNA expression after transient transfection with different siRNA fragments (*n* = 3). ^*∗*^*P* < 0.05 and ^*∗∗*^*P* < 0.01, compared with siRNA-NC group, as assessed by paired Student's *t*-test. (c) Western blot to detect Cdc42 protein expression after transient transfection with different siRNA fragments (*n* = 3). ^*∗∗*^*P* < 0.01, compared with siRNA-NC group, as assessed by paired Student's *t*-test. (d) Effects of Cdc42 on MIN6 cell proliferation. CCK assay to detect MIN6 cell proliferation after transfection with Cdc42-siRNA and Cdc42-pcDNA3.1 (*n* = 3). ^*∗∗*^*P* < 0.01, compared with siRNA-NC group, ^#^*P* < 0.01, compared with pcDNA3.1 group, as assessed by one-way ANOVA, followed by Fisher's least significant difference test. (e and f) Effects of Cdc42 on insulin secretion by MIN6 cells. ELISA to detect insulin secretion levels in MIN6 cells after transient transfection with Cdc42-pcDNA3.1 and Cdc42-siRNA under basal-glucose (5.0 mM) and high-glucose (20.0 mM) stimulation (*n* = 3). ^*∗*^*P* < 0.05, compared with pcDNA3.1 group, ^*∗∗*^*P* < 0.01, compared with siRNA-NC group, and ^##^*P* < 0.01, compared with 5.0 mM glucose group, as assessed by paired Student's *t*-test. Data are shown as mean ± SD. NC: negative control.

To ensure the validity of subsequent Cdc42-related experiments, Cdc42-pcDNA3.1 was transiently transfected into MIN6 cells when the cell confluency reached 75%, and protein extraction was carried out after 40 h. The western blot results showed that Cdc42 expression increased after transfection with Cdc42-pcDNA3.1 compared with the expression in the pcDNA3.1 group (*P* < 0.01) (Figure 2(a)). This result indicated successful transfection.

#### 3.2.2. Screening of Cdc42 Small Interfering RNA (siRNA) Fragments

To effectively reduce the Cdc42 expression, we screened three siRNA fragments (siRNA-497, siRNA-569, and siRNA-643) to identify which one was the most effective. When the cell confluency reached 75%, NC-siRNA, siRNA-497, siRNA-569, and siRNA-643 were transiently transfected into MIN6 cells. In each group, total RNA was extracted after 36 h. The RT-PCR results showed that Cdc42 mRNA expression significantly decreased after transfection with siRNA-497 (*P* < 0.05) and siRNA-643 (*P* < 0.01) compared with the expression in the siRNA-NC group. Among the four groups, the siRNA-643 group had the lowest Cdc42 mRNA expression (Figure 2(b)). The results indicated that siRNA-497 and siRNA-643 could more effectively reduce Cdc42 mRNA expression than siRNA-569, and siRNA-643 may have the optimal interference effect.

To determine whether siRNA-643 was the optimal siRNA fragment, when the cell confluency reached 75%, the siRNAs were transiently transfected into MIN6 cells, and protein extraction was carried out after 40 h. The western blot results showed that Cdc42 protein expression in the siRNA-569 and siRNA-643 groups significantly decreased (*P* < 0.01) compared with the expression in the siRNA-NC group. Among the four groups, the siRNA-643 group had the lowest Cdc42 protein expression (Figure 2(c)). Based on the Cdc42 mRNA and protein expression levels, we selected siRNA-643 as the Cdc42-siRNA fragment to use in subsequent experiments.

#### 3.2.3. Cdc42 Positively Effects MIN6 Cell Proliferation

To identify the effect of Cdc42 on MIN6 cells proliferation, the absorbance at 450 nm was measured at 24, 48 and 72 h after transient transfection of MIN6 cells with Cdc42-pcDNA3.1 and Cdc42-siRNA-643, and corresponding cell growth curves were plotted. The CCK results showed that there were no significant differences in the proliferation rate between the Cdc42-pcDNA3.1 and pcDNA3.1 groups, or between the Cdc42-siRNA-643 and siRNA-NC groups, after 24 h. In contrast, the proliferation rate in the Cdc42-siRNA-643 group was significantly decreased after 48 h (*P* < 0.01) and 72 h (*P* < 0.01), and the proliferation rate in the Cdc42-pcDNA3.1 group was significantly increased after 48 h (*P* < 0.01) and 72 h (*P* < 0.01) (Figure 2(d)). These results indicated that Cdc42 positively effects the proliferation rate of MIN6 cells.

#### 3.2.4. Cdc42 Positively Effects Insulin Secretion by MIN6 Cells

To identify the effect of Cdc42 on insulin secretion by MIN6 cells, we increased and decreased Cdc42 expression using Cdc42-pcDNA3.1 and Cdc42-siRNA-643, respectively, and detected the level of insulin secretion under 5.0 and 20.0 mM glucose stimulation by measuring the amount of secreted insulin in the supernatant. The ELISA results showed that Cdc42 overexpression promoted insulin secretion under high-glucose stimulation (*P* < 0.05) (Figure 2(e)), and Cdc42 downregulation inhibited insulin secretion under high-glucose stimulation (*P* < 0.01) (Figure 2(f)). Regardless of whether Cdc42 was up- or downregulated, there were no effects on insulin secretion under basal-glucose stimulation (Figures 2(e) and 2(f)). These results indicated that Cdc42 plays a positive regulatory role in GSIS, but not in insulin secretion at physiological blood glucose levels.

### 3.3. Effects of miR-29a/Cdc42 on MIN6 Cells

#### 3.3.1. miR-29a Negatively Effects Cdc42 Protein Expression

**Figure 3:**
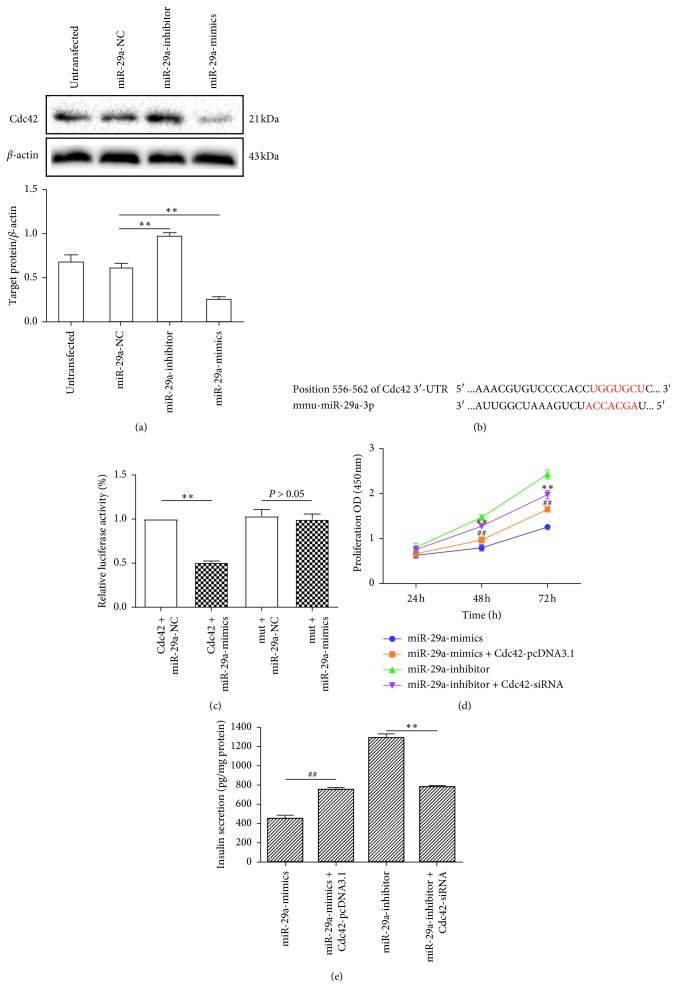
Effects of miR-29a/Cdc42 on MIN6 cells. (a) Effects of miR-29a on Cdc42 protein expression. Western blot to detect Cdc42 protein expression after transfection with miR-29a mimic and inhibitor (*n* = 3). ^*∗∗*^*P* < 0.01, compared with miR-29a NC group, as assessed by paired Student's *t*-test. (b) Bioinformatics analysis of the miR-29a binding site in the Cdc42 mRNA 3′-UTR. (c) Luciferase reporter assays indicate that miR-29a binds to Cdc42 mRNA in MIN6 cells. ^*∗∗*^*P* < 0.05, compared with nonmutated Cdc42 3′UTR + miR-29a-NC group, as assessed by paired Student's *t*-test. (d) Effects of miR-29a/Cdc42 on MIN6 cell proliferation. CCK assay to detect MIN6 cell proliferation after simultaneous overexpression of miR-29a and Cdc42, and after simultaneous interference with miR-29a and Cdc42 expression (*n* = 3). ^*∗∗*^*P* < 0.01, compared with miR-29a mimic group, and ^##^*P* < 0.01, compared with miR-29a inhibitor group, as assessed by one-way ANOVA, followed by Fisher's least significant difference test. (e) Effects of miR-29a/Cdc42 on insulin secretion by MIN6 cells. ELISA to detect insulin secretion by MIN6 cells after transient transfection with miR-29a mimic + Cdc42-pcDNA3.1 and miR-29a inhibitor + Cdc42-siRNA under high-glucose (20.0 mM) stimulation (*n* = 3). ^*∗∗*^*P* < 0.01, compared with miR-29a inhibitor group, and ^##^*P* < 0.01, compared with miR-29a mimic group, as assessed by paired Student's *t*-test. Data are shown as mean ± SD. NC: negative control; mmu: mouse lemur; 3p: mmu-miR-29a is produced from the 3′ end arm of the double strand of miR-29a; mut: mutated.

Many studies have indicated that Cdc42 mRNA is a direct target of miR-29a in cancer progression [42–46]. Thus, we hypothesized that miR-29a can affect the expression of Cdc42 during diabetes progression. To identify the effect of miR-29a on Cdc42 protein expression, we transiently transfected the miR-29a mimic, miR-29a inhibitor, and miR-29a-NC into MIN6 cells when the cell confluency reached 75%, and extracted the proteins for each group after 40 h. The western blot results showed that, compared with the Cdc42 protein expression in the miR-29a NC group, the expression in the miR-29a mimic group significantly decreased (*P* < 0.01), whereas the expression in the miR-29a inhibitor group significantly increased (*P* < 0.01) (Figure 3(a)). These results indicated that miR-29a negatively effects Cdc42 protein expression.

#### 3.3.2. miR-29a Binding Site in the Cdc42 mRNA 3′-UTR

Based on the negative effect of miR-29a on Cdc42 protein expression and in order to confirm that Cdc42 mRNA is a target of miR-29a, the miR-29a binding site in the Cdc42 mRNA 3′-UTR was identified in a bioinformatics analysis and a luciferase reporter assay was performed (Figures 3(b) and 3(c)). The bioinformatics analysis showed that the Cdc42 mRNA 3′-UTR was targeted by the complementary sequence of miR-29a (Figure 3(b)). The luciferase reporter assays showed that the miR-29a mimic significantly decreased the luciferase activity of MIN6 cells expressing the nonmutated Cdc42 mRNA 3′-UTR, but it had no effect on the luciferase activity of MIN6 cells expressing the mutated Cdc42 mRNA 3′-UTR (Figure 3(c)). These results showed that Cdc42 mRNA is a potential downstream molecule of miR-29a, and the miR-29a/Cdc42 axis may be involved in diabetes.

#### 3.3.3. Effects of miR-29a/Cdc42 on MIN6 Cell Proliferation

To identify the effects of miR-29a/Cdc42 on MIN6 cell proliferation, we transfected miR-29a inhibitor + Cdc42-siRNA-643 and miR-29a mimic + Cdc42-pcDNA3.1 into MIN6 cells. The absorbance at 450 nm was measured at 24, 48, and 72 h after transient transfection, and corresponding cell growth curves were plotted. The CCK results showed that after 48 and 72 h, simultaneous overexpression of miR-29a and Cdc42 reversed the effect of the miR-29a mimic regarding MIN6 cell proliferation inhibition (*P* < 0.01). Simultaneous interference with miR-29a and Cdc42 expression reversed the effect of the miR-29a inhibitor regarding MIN6 cell proliferation promotion (*P* < 0.01) (Figure 3(d)). These results further illustrated that Cdc42 mRNA is a downstream molecule of miR-29a and indicated that miR-29a can inhibit MIN6 cell proliferation by downregulating Cdc42 expression.

#### 3.3.4. Effects of miR-29a/Cdc42 on Insulin Secretion by MIN6 Cells

To identify the effects of miR-29a/Cdc42 on insulin secretion by MIN6 cells, we transiently transfected miR-29a inhibitor + Cdc42-siRNA-643 and miR-29a mimic + Cdc42-pcDNA3.1 into MIN6 cells, and then stimulated them with KRBH containing 20.0 mM glucose for 1 h. The amount of secreted insulin in the supernatant was measured by ELISA. The results showed that simultaneous overexpression of miR-29a and Cdc42 reversed the effect of the miR-29a mimic regarding insulin secretion inhibition (*P* < 0.01), and simultaneous interference with miR-29a and Cdc42 expression reversed the effect of the miR-29a inhibitor regarding the promotion of insulin secretion under high-glucose stimulation (*P* < 0.01) (Figure 3(e)). These results further indicated that miR-29a can inhibit insulin secretion by MIN6 cells under high-glucose stimulation by downregulating Cdc42 expression.

### 3.4. miR-29a/Cdc42/*β*-Catenin is a Potential Signaling Cascade in MIN6 Cells

**Figure 4:**
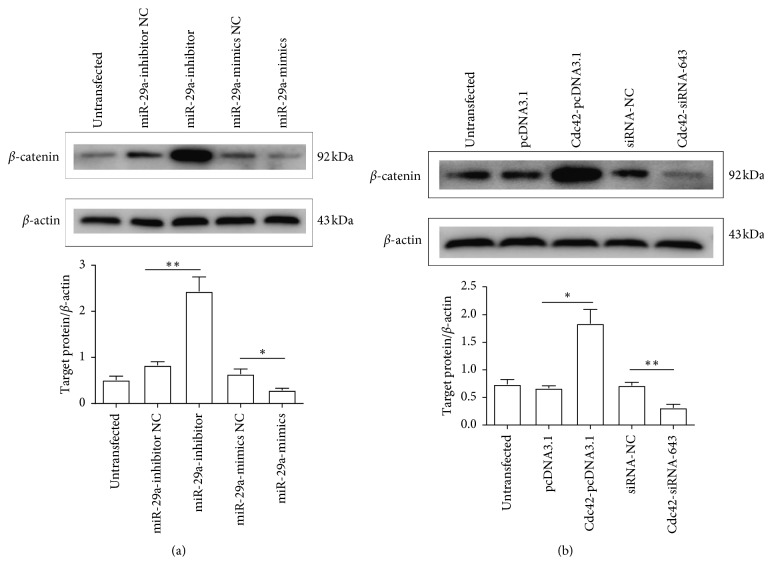
Effects of miR-29a and Cdc42 on *β*-catenin expression. (a) Effects of miR-29a on *β*-catenin expression. Western blot to detect *β*-catenin expression after transfection with miR-29a mimic and inhibitor (*n* = 3). ^*∗*^*P* < 0.01, compared with miR-29a mimic NC, and ^*∗∗*^*P* < 0.01, compared with miR-29a inhibitor NC, as assessed by paired Student's *t*-test. (b) Effects of Cdc42 on *β*-catenin expression. Western blot to detect *β*-catenin expression after transfection with Cdc42-pcDNA3.1 and siRNA-643 (*n* = 3). ^*∗*^*P* < 0.01, compared with pcDNA3.1, and ^*∗∗*^*P* < 0.01, compared with siRNA-NC, as assessed by paired Student's *t*-test. Data are shown as mean ± SD. NC: negative control.

Many studies have demonstrated that *β*-catenin expression can be regulated by Cdc42 [47–49]. Therefore, we hypothesized that *β*-catenin is a downstream molecule of miR-29a/Cdc42, and miR-29a/Cdc42/*β*-catenin is a potential signaling cascade in MIN6 cells. We transfected miR-29a mimic, miR-29a inhibitor, Cdc42-pcDNA3.1, and Cdc42-siRNA-643 into MIN6 cells. The western blot results showed that compared with *β*-catenin expression in the miR-29a mimic and inhibitor NC, miR-29a downregulation significantly increased *β*-catenin expression (*P* < 0.01), whereas miR-29a overexpression significantly decreased *β*-catenin expression (*P* < 0.01) (Figure 4(a)). Conversely, Cdc42 overexpression significantly increased *β*-catenin expression (*P* < 0.01), whereas Cdc42 downregulation significantly decreased *β*-catenin expression (*P* < 0.01) (Figure 4(b)). These results indicated that miR-29a can inhibit but Cdc42 can promote *β*-catenin protein expression in MIN6 cells.

## 4. Discussion

Apart from being one of the leading causes of death worldwide, hyperglycemia in diabetic patients endangers microvessel in various target organs; for example, the brain, heart, kidneys, and eyes [50]. Proliferation impairment of *β* cells is the main cause of T1DM and GSIS impairment in *β* cells is the main cause of T2DM [1, 31, 51]. Therefore, it is important to understand the underlying mechanisms that affect proliferation and GSIS of *β* cells in order to improve the development of pharmacological agents for diabetes treatment. In most cases, miRNAs act as negative regulators and affect protein-coding genes; therefore, abnormal miRNA expression interferes a variety of physiological and pathophysiological processes, including insulin secretion. Many studies have shown that Cdc42 is an important regulatory gene in GSIS [25], via activating its downstream effector p21-activated kinase (Pak1; a Ser/Thr protein kinase) during the second phase of GSIS [52–54]. Cdc42 locates on insulin secretory granules, and it participates in exocytosis of insulin vesicles by regulating F-actin and its associated pathways [55].

In this study, the MIN6 cell line was selected to investigate cell proliferation and insulin secretion *in vitro* for the following four reasons: (a) the MIN6 cell line was established from pancreatic tumors of transgenic non-obese diabetic mice, and the insulin secretory function of MIN6 cells is highly similar to that of the pancreas; (b) islet *β* cells are the most abundant cells among islet cells, accounting for about 70% of the total; (c) the insulin secretory function of MIN6 cells is highly similar to that of the pancreas; and (d) most of the related studies in the literature used this cell line to study insulin secretion and related signaling pathways, so the body of literature on MIN6 cells is relatively rich. Both T1DM and T2DM involve insulin deficiency, and T1DM also involves loss of *β* cells, so the study of the role of miR-29a/Cdc42/*β*-catenin in MIN6 cell proliferation and GSIS will be helpful to explore the molecular mechanisms of T1DM and T2DM. Our experiment first explored effects of miR-29a and Cdc42, and the mechanism of miR-29a/Cdc42/*β*-catenin pathway on MIN6 cells proliferation and GSIS under high-glucose condition.

The CCK and ELISA results showed that miR-29a inhibits MIN6 cell proliferation and insulin secretion under high-glucose stimulation, but there were no significant differences under basal-glucose stimulation. This result is consistent with the results of an in vitro study by Bagge et al. [24] on the INS-1E cell line, but it conflicts with the conclusions of an in vivo study by Dooley et al. [20],in which miR-29a/b-1 was knocked out because it was not possible to knockout only miR-29a. Thus, these conflicting results may be caused by knocking out miR-29b-1 or by the differences between the *in vitro* and *in vivo* approaches.

The inhibition of Cdc42 protein expression using siRNA-569 appeared to be as effective as that using siRNA-643. Compared with the siRNA-NC group, the inhibition of Cdc42 mRNA expression using siRNA-569 was also effective, but there was no statistical difference. The possible reason was that our experiment was only performed three times. And siRNA-643 showed the best inhibitory effect on Cdc42 mRNA and protein expression among the screened siRNA fragments; therefore, siRNA-643 was used for subsequent experiments. The CCK and ELISA results showed that Cdc42 promotes MIN6 cell proliferation and insulin secretion under high-glucose stimulation, but there was no significant difference under basal-glucose stimulation. The results of this experiment are consistent with current mainstream thinking in this field. Current researchers generally believe that Cdc42 plays a positive regulatory role in GSIS and interfering with its expression decreases insulin secretion [25]. Cdc42 can affect insulin secretion by regulation of insulin vesicle fusion, exocytosis, and cytoskeletal rearrangement [56], but the specific pathways involved in this process require further research.

In addition, we investigated whether miR-29a affects MIN6 cell proliferation and insulin secretion by interfering with Cdc42 expression. The western blot results showed that miR-29a has a negative regulatory role regarding Cdc42 expression in MIN6 cells, which is similar to the findings for cancers such as nonsmall-cell carcinoma, stomach cancer, and breast cancer [42, 43, 57]. Furthermore, the bioinformatics analysis and luciferase reporter assay showed that there is a miR-29a binding site in the Cdc42 mRNA 3′-UTR, and miR-29a can therefore affect the expression of Cdc42 and downstream molecules, ultimately exerting biological effects. And Cdc42 can reverse the effects of miR-29a on MIN6 cell proliferation and GSIS under high-glucose condition. It is worth mentioning that regardless of whether miR-29a or Cdc42 was up- or downregulated, there were no effects on GSIS under basal-glucose stimulation; therefore, effects of miR-29a/Cdc42 on MIN6 cells GSIS under basal-glucose stimulation study seems less necessary. These results suggest that Cdc42 is a direct effector of miR-29a *in vitro*, and miR-29a can suppress MIN6 cells proliferation and GSIS via negatively regulating Cdc42 expression.

Many studies have demonstrated that *β*-catenin can be regulated effectively by Cdc42 [47–49], and we hypothesized that miR-29a/Cdc42/*β*-catenin is a potential signaling cascade involved in diabetes progression. Collectively, miR-29a can negatively affect the expression of Cdc42 and downstream molecule *β*-catenin, and, therefore, suppress F-actin remodeling, insulin granules mobilization, and cell-to-cell interaction, and ultimately inhibit GSIS by MIN6 cells [39, 58]. Besides, a low *β*-catenin protein expression may inhibit *β*-catenin nuclear translocation and cyclins D1, D2, and c-Myc gene expression, so miR-29a can negatively affect the proliferation rate by MIN6 cells [59]. Therefore, upregulation of miR-29a associated with inhibition of Cdc42/*β*-catenin signaling may be potential factors in MIN6 cells proliferation and GSIS suppression.

## 5. Conclusions

In conclusion, the current study reports the role of miR-29a in MIN6 cell proliferation and GSIS, which involves regulating Cdc42 and *β*-catenin expression. The results indicate that miR-29a inhibits MIN6 cells proliferation and GSIS and negatively regulates Cdc42 expression. In contrast, Cdc42/*β*-catenin is a miR-29a downstream signaling that promotes MIN6 cells proliferation and GSIS. To summarize, miR-29a can negatively affect GSIS and MIN6 cell proliferation via Cdc42/*β*-catenin signaling. miR-29a/Cdc42/*β*-catenin may be involved in diabetes progression. However, further animal experiments and studies of clinical samples from patients are needed to validate the function of miR-29a/Cdc42/*β*-catenin, and whether other miRNAs and downstream molecules play crucial roles in diabetes progression also requires further studies.

## Figures and Tables

**Table 1:** The sequences of miR-29a mimic and Cdc42-mus.

Name	Sequences (5′-3′)
miRNA-29a mimic	UAGCACCAUCUGAAAUCGGUUA ACCGAUUUCAGAUGGUGCUAUU
Cdc42-mus-643	UCACACAGAAAGGCCUAAATT UUUAGGCCUUUCUGUGUGATT
Cdc42-mus-569	GCCUAUUACUCCAGAGACUTT AGUCUCUGGAGUAAUAGGCTT
Cdc42-mus-497	GCUUGUUGGGACCCAAAUUTT AAUUUGGGUCCCAACAAGCTT

## Data Availability

The data used to support the findings of this study are available from the corresponding author upon request.
